# Dogs as Reservoirs for *Leishmania braziliensis*

**DOI:** 10.3201/eid1702.091823

**Published:** 2011-02

**Authors:** Filipe Dantas-Torres

**Affiliations:** Author affilion: Università degli Studi di Bari, Bari, Italy

**To the Editor**: I have read the review by Sousa and Pearson (*1*), which provides a fascinating historical account of the Great Drought and the smallpox epidemic of the 1870s and their association with the emergence of cutaneous leishmaniasis in Ceará, Brazil. In their review, the authors went back to the 19th century, remembering the hard years experienced by those who faced the Great Drought, which prompted the immigration of thousands of persons from Ceará to the Amazon region, and a devastating smallpox epidemic, which resulted in the death of >100,000 persons. Later, they returned to the present situation of cutaneous leishmaniasis in Brazil.

I would like to address the role of dogs as reservoirs of *Leishmania* (*Viannia*) *braziliensis*. Sousa and Pearson stated that “no animal reservoir other than dogs has been identified in Ceará” and that “a sylvatic reservoir has not been identified for *L.* (*V.*) *braziliensis* in Ceará and other areas,” concluding that “dogs appear to be the most important reservoir in domestic and peridomestic transmission.”

Conversely, recent studies have indicated that rodents and other small mammals are the primary reservoirs for *L.* (*V.*) *braziliensis* (*2*) and that, so far, no strong evidence indicates that dogs could act as reservoirs for this parasite (*3**,**4*). The finding of dogs infected by *L.* (*V.*) *braziliensis* in leishmaniasis-endemic areas is expected because they are susceptible to this parasite and are often exposed to phlebotomine sandflies. However, this finding does not imply that dogs are important reservoirs. Indeed, they represent a poor source of *L.* (*V.*) *braziliensis* (*3*). For these reasons, dogs cannot be incriminated as the most important reservoirs in the domestic and peridomestic transmission cycles of *L.* (*V.*) *braziliensis*.
