# Supplementary material for: Dogs as Reservoirs for Leishmania braziliensis
Source: Emerg Infect Dis. 2011 Feb;17(2):326–7. doi: 10.3201/eid1702.091823 (PMC3379898; doi:10.3201/eid1702.091823)
Supplement: Related texts

# Reply

**DOI:** 10.3201/eid1702.101669

**Authors:** Anastácio de Queiroz Sousa, Richard D. Pearson

**Affiliations:** Author affiliations: Federal University of Ceará School of Medicine, Fortaleza, Brazil (A.Q. Sousa);; University of Virginia School of Medicine, Charlottesville, Virginia, USA (R.D. Pearson)

**Keywords:** *Suggested citation for this article*: de Queiroz Sousa A, Pearson RD. Dogs as reservoirs for *Leishmania braziliensis* [letter]. Emerg Infect Dis [serial on the Internet]. 2011 Feb [*date cited*]. http://dx.doi.org/10.3201/eid1702.101669

**In Response:** We agree with most points raised by Dantas-Torres (*1*). However, so far our statement related to the reservoir(s) of *Leishmania* (*Viannia*) *braziliensis* (*2*) is correct for Ceará. Oliviera-Lima (*3*) conducted a case–control study that definitively incriminated dogs as a reservoir host of *L.* (*V.*) *braziliensis* in Baturite, Ceará. He showed that infected dogs in households increased the risk for infection with *L.* (*V.*) *braziliensis* by 3.2-fold among resident children. The risk increased to 6-fold when infected dogs had leishmaniasis-like skin lesions. Additionally, his observations suggested that animals other than dogs might be involved. In another study, Santana (*4*) examined 272 small mammals (213 rodents and 59 marsupials) in the same area; tissue culture and hamster inoculation showed none to be infected with *L.* (*V.*) *braziliensis*, although some cultures were contaminated by fungi and bacteria. On the basis of these findings, a sylvatic reservoir for *L.* (*V.*) *braziliensis* has not been identified in Baturite, Ceará. However, Brandão-Filho et al. found that rodents in Pernambuco State, Brazil, were infected with *L.* (*V.*) *braziliensis* and stated that they were the primary reservoir (*5*). We concur with Lainson and Shaw (*6*) and conclude that information about mammalian reservoirs of *L.* (*V.*) *braziliensis* is incomplete.

## References

[1] Dantas-Torres F. Dogs as reservoirs for *Leishmania braziliensis* [letter]. Emerg Infect Dis. 2011;17:326–7.2129162610.3201/eid1702.091823PMC3379898

[2] Sousa AQ, Pearson R. Drought, smallpox, and emergence of *Leishmania braziliensis* in northeastern Brazil. Emerg Infect Dis. 2009;15:916–21. 10.3201/eid1506.07133119523291PMC2727349

[3] Oliveira-Lima JW. Domestic transmission of cutaneous leishmaniasis in Brazil [dissertation]. Cambridge (MA): Harvard University; 1995.

[4] Santana EW. The role of small mammals as reservoir hosts of cutaneous leishmaniasis in the “serra de Baturité,” an endemic zone in Ceará State, Brazil [dissertation]. Bristol (UK): University of Bristol; 1999.

[5] Brandão-Filho SP, Brito ME, Carvalho FG, Ishikawa EA, Cupolillo E, Floeter-Winter L, Wild and synanthropic hosts of *Leishmania* (*Viannia*) *braziliensis* in the endemic cutaneous leishmaniasis locality of Amaraji, Pernambuco State, Brazil. Trans R Soc Trop Med Hyg. 2003;97:291–6. 10.1016/S0035-9203(03)90146-515228244

[6] Lainson R, Shaw JJ. Evolution, classification and geographical distribution. In: Peters W, Killick-Kendrick R, editors. The leishmaniases in biology and medicine. Vol. 1. London: Academic Press; 1987. p. 1–120.
